# The p(l)ot thickens: cannabinoid receptors on astroglial mitochondria coordinate animal behaviors by regulating lactate availability for neurons

**DOI:** 10.1038/s41392-020-00298-1

**Published:** 2020-09-03

**Authors:** Erik Keimpema, Tibor Harkany, Alán Alpár

**Affiliations:** 1 Department of Molecular Neurosciences, Center for Brain Research, Medical University of Vienna, Vienna, Austria; 2 Department of Neuroscience, Biomedicum D7, Karolinska Institutet, Solna, Sweden; 3 SE NAP Research Group of Experimental Neuroanatomy and Developmental Biology, Semmelweis University, Budapest, Hungary; 4 Department of Anatomy, Histology, and Embryology, Semmelweis University, Budapest, Hungary

**Keywords:** Cellular neuroscience, Diseases of the nervous system, Metabolic disorders

The coordinated interplay between neurons and astroglia, the latter producing essential metabolic precursors for neurons, is critical for the precision of neurotransmission in the execution of specific behaviors. Most recently, Jimenez-Blasco et al.^1^ identified CB_1_ cannabinoid receptors in astroglial mitochondria as gatekeepers of lactate production by regulating the stability of mitochondrial complex I through the hypoxia-inducible factor-1 pathway and thus, metabolically tuning neurons that control social behaviors.

The desire to understand how neurons and (astro-)glia interact to orchestrate specific behaviors is a focal point of neuroscience research. Equally, impairment of neuron-glia communication is increasingly linked to neuropsychiatric anomalies, including responses to psychoactive drugs. Therefore, mapping molecular constituents, particularly receptors and signal transduction pathways that partition to or become enriched in ‘tripartite synapses’ (that is, pre- and postsynaptic termini and ensheathing astroglia) continues to generate significant interest.

A key example of linking psychoactive drug action to receptor function is the discovery of the CB_1_ cannabinoid receptor (CB_1_R), which is the primary binding site for Δ^9^-tetrahydrocannabinol (THC) from marijuana.^2^ An avalanche of histochemical studies lifted CB_1_Rs from being an isolated molecular feature of some GABAergic interneurons to being recognized as one of the most abundant G protein-coupled receptors (GPCRs) in the brain. Thus, the existence of CB_1_Rs to modulate neurotransmission became the rule, rather than the exception, at central synapses.

The classical concept of GPCR-mediated signal transduction posits receptors at the cell surface with signaling cascades operating in the cytosol to change cellular states. This arrangement was revised by the identification of intracellular receptors. The particular GPCR subclasses for which lipophilic ligands can be equally efficacious at both the cell surface and intracellularly provide exciting cases to dissociate the significance of subcellular receptor partitioning. In this context, a breakthrough in the study of (endo-)cannabinoid signaling came with the discovery that CB_1_Rs can accumulate in neuronal mitochondria (mtCB_1_Rs) to limit respiration, thus dampening synaptic communication, a concept compatible with the amnesic effects of THC.^3,4^

However, the above neuron-centric view is about to change. This is because the team led by Giovanni Marsicano has gone beyond many thoughts relevant and characterized mtCB_1_Rs in astroglia,^1^ a receptor contingent relegated to ‘background signal’ for most of the past decade. They combined molecular biology, pharmacology, biochemistry and mouse genetics to link astroglial mtCB_1_Rs to glycolysis and lactate production for subsequent uptake by neurons and their utilization to sustain specific behaviors.

**Fig. 1:**
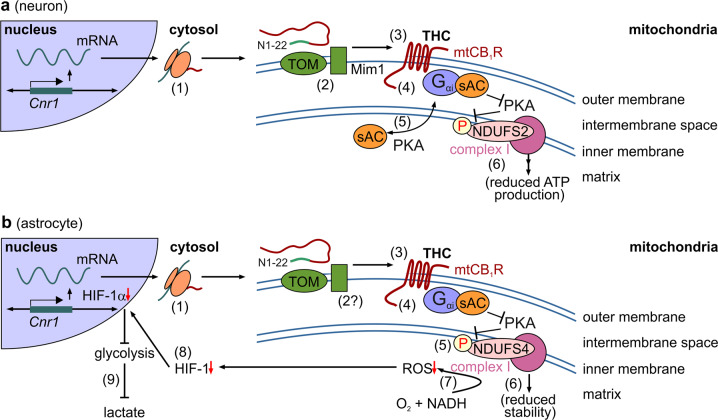
Comparison of signaling cascades induced by mtCB1R activation in neurons and astrocytes. **a** In neurons,^3^ a pool of CB_1_Rs (1) is targeted to mitochondria (‘mtCB_1_Rs’). Even though the N1-22 sequence facilitates mitochondrial enrichment, the exact mitochondrial import machinery for CB_1_Rs is as yet debated (2). mtCB1Rs localize to the outer mitochondrial membrane.^3^ Δ^9^-tetrahydrocannabinol (THC) action on mtCB1Rs (3) reduces cellular ATP availability through a mechanism involving inhibition of soluble adenylate cyclase (sAC) (4) and subsequent inhibition of the PKA-dependent phosphorylation of complex I proteins, particularly NDUFS2 (6). An open question is the precise partitioning of signal proteins (5). Data were adapted from ref. ^4^ **b** In astrocytes,^1^ the mitochondrial import of CB_1_Rs is elusive yet could follow similar principles as in neurons (1,2). mtCB_1_Rs, when activated (3), reduce the protein kinase A (PKA)-dependent phosphorylation of the mitochondrial complex I subunit NDUFS4 (4,5). NDUFS4 hypophosphorylation destabilizes complex I (6), including a detrimental effect on its ability of producing reactive oxygen species (ROS, 7). Reduced ROS levels, in turn, impair HIF-1 signaling (7) and HIF-1-dependent transcription (8), ultimately leading to an HIF-1α-dependent reduction of lactate production (9)

The newest study^1^ by the Marsicano group is exemplary in detail, versatility and comparison of the role of astroglial mtCB_1_Rs with earlier data in neurons (Fig. 1a).^3,4^ First, mtCB_1_Rs in astroglia were suggested as a brain-wide phenomenon by being present in the nucleus accumbens, hippocampus, piriform, and prefrontal cortices. Activation of mtCB_1_Rs, alike in neurons,^3^ reduced astrocytic O_2_ consumption in a soluble adenylyl cyclase-dependent manner. A key advance came from the introduction of tamoxifen-inducible astroglia-specific knock-out mice (using a *Gfap*-CreER^T2^ driver), which showed a proportionate response when compared to *Cnr1*^−/−^ (null) mice.

A particularly impressive aspect of this work is the biochemical dissection of how mtCB_1_R activity destabilizes mitochondrial complex I in astrocytes (Fig. 1b): mtCB_1_R activation (including by THC) reduces complex I activity, which is critical for NADH binding and oxidation. This is through the disruption of its N- but not P-module (as shown by decreased NDUFS1/NDUFV2 levels). THC-induced mtCB_1_R activation reduced the phosphorylation of complex I proteins, notably of NDUFS4 (at its Ser^173^ residue), which is critical for the assembly and stability of complex I. An exemplary series of in vitro and in vivo controls with phosphomimetic point-mutant constructs, and cell type-specific viral and transgene technologies reinforced the critical role of reducing NDUFS4 phosphorylation at Ser^173^ as detrimental for astrocytes and, more broadly, that cannabinoids are efficacious to destabilize astrocytic energy metabolism specifically through mtCB_1_Rs.

A key physiological role of the N-module of complex I is through its O_2_ binding pocket where mitochondrial reactive oxygen species (mROS) are produced upon acceptance of electrons from NADH(H^+^). Accordingly, a destabilized complex I shall lead to reduced mROS levels. As predicted, activation of astrocytic mtCB_1_Rs by THC diminished mROS levels in vitro, an effect being both CB_1_R dependent and negated by reinstating NDUFS4 activity. The hypoxia-inducible factor 1 (HIF-1) transcriptional cascade chiefly detects mROS in astrocytes. Indeed, nuclear HIF-1 levels, as well as its promoter activity were reduced by THC, an observation completing the mechanistic definition of a mtCB_1_R–complex I–mROS–HIF-1 cascade. As HIF-1 signaling physiologically stimulates glycolysis, it is logical that THC reduces glycolytic activity and, ultimately, lactate production in astrocytes.

The uptake of astroglia-derived lactate through monocarboxylate transporters (particularly MCT2) by neurons is critical for the maintenance of neuronal bioenergetics. Thus, an impaired astroglia-to-neuron lactate shuttle will have dire consequences on neuronal function. Indeed, the THC-induced reduction in lactate availability provoked neuronal death and affected mtCB_1_R-dependent behaviors, alike reported for neuronal mtCB_1_Rs earlier.^3,4^ Nevertheless, this study makes a leap forward by specifically linking THC action through astrocytic mtCB_1_Rs to impaired social interaction, at least in mice. The finding that other cannabinoid-sensitive behaviors (e.g., locomotion, anxiety) remained unchanged provoke the question if and how task-specific (mt)CB_1_R recruitment and engagement could occur in a cell type-specific manner in the brain.

While this is an entirely experimental study, its human relevance might be significant, including future cancer therapy. Tumors are the most frequent and robust examples of glial dysfunction. By switching to aerobic glycolysis, glioblastoma cells produce vast amounts of lactate, known as the Wartburg effect. This decreases extracellular pH and promotes angiogenesis and metastasis.^5^ A key player in this process is lactate dehydrogenase, whose impinging on neuronal glutamate signaling compromises neuronal function and triggers apoptosis in neighboring brain tissue. As lactate dehydrogenase inhibitors do not cross the blood-brain-barrier, the golden standard of glioblastoma treatment is glycolytic inhibition and a ketogenic diet to induce metabolic oxidative stress in cancer cells, making them more susceptible to chemotherapy, radiotherapy, and immunotherapy. As mtCB_1_Rs decrease lactate availability, administration of CB_1_R agonists could slow glioblastoma growth and limit nearby tissue degeneration. Moreover, glioblastoma cells can receive glutamatergic synapses that, through Ca^2+^ signaling coordinated in small-world glioma cell assemblies can promote tumor invasion. Thus, dampening neuronal activity, whether directly at neuronal cell-surface CB_1_Rs or by disrupting lactate production by glioblastoma could procure a ‘dual-hit’ profile for cannabinoid therapies. This concept accords with beneficial cannabinoid effects (including THC) in inhibiting glioblastoma growth and invasion in both animal models and human trials.^6^ Furthermore, mitochondrial upregulation of HIF-1 has been shown to reduce the efficacy of temozolomide treatment, a therapy blocking gene transcription to weaken tumor cells. Reducing HIF-1 with cannabinoid treatment, therefore, could account for reduced lactate production in synchrony with improved HIF-1 targeting. Overall, these considerations lift the study above and beyond an exemplary description of glia-to-neuron communication by potentially pinpointing key molecular targets that are of paramount importance for both brain physiology and pathology.
